# ASSESSING AGE INCLUSIVITY USING THE AGE-FRIENDLY INVENTORY AND CAMPUS CLIMATE SURVEY

**DOI:** 10.1093/geroni/igae098.1588

**Published:** 2024-12-31

**Authors:** Jodi Waterhouse

**Affiliations:** CU Anschutz Multidisciplinary Center on Aging, Aurora, Colorado, United States

## Abstract

University of Colorado Anschutz Medical Campus became designated as an Age-Friendly Academic Medical Campus in 2021. However, the campus is part of a much larger campus community which includes a large health system, a children’s hospital, and a VA Medical Center, that are not Age-Friendly designated. More than 30,000 employees, visitors, and vendors visit the Anschutz campus daily. How does an academic medical campus, surrounded by three large health system hospitals and clinics ensure that visiting is easy, enjoyable, and accessible, and visitors don’t get lost in other campus partner’s buildings? The Age-Friendly Inventory and Campus Climate Survey helped provide the framework for guidance to identify issues including parking, parking payment kiosks, signage, and campus maps both virtual and print. This session will provide how a large academic medical campus and its representatives were able to identify and personally experience problematic hurdles when visiting a large university campus; what Built Environment Assessment experience was collected to identify issues; and how recommendations for improvement were presented to and supported by campus leadership.

